# Guideline concordant screening and monitoring of extrapyramidal symptoms in patients prescribed antipsychotic medication: a systematic review and narrative synthesis

**DOI:** 10.1192/j.eurpsy.2025.455

**Published:** 2025-08-26

**Authors:** R. Aubry, T. Hastings, M. Morgan, J. Hastings, M. Bolton, M. Grummell, R. Shorr, S. Killeen, C. Coyne, M. Solmi

**Affiliations:** 1 Psychiatry, Lucena Clinic Services, St John of God Hospital, Dublin, Ireland; 2 Psychiatry, University of Toronto, Toronto, Canada; 3 Psychiatry, HSE CAMHS, Drogheda; 4 School of Medicine, University College Dublin; 5 Psychiatry, St Michael’s House, Services for people with disabilities; 6 Psychiatry, HSE Phoenix Care Centre, Dublin, Ireland; 7 Learning Services, The Ottawa Hospital, Ottawa, Canada; 8 West Kildare CAMHS, Abbeylands Clane, Ireland; 9 Clinical Epidemiology Program, Ottawa Hospital Research Institute, Ottawa, Canada

## Abstract

**Introduction:**

Given the increasing rates of antipsychotic use in multiple psychiatric conditions, greater attention to the assessment, monitoring and documentation of their side effects is warranted. While significant attention has been provided to metabolic side effect monitoring, comparatively little is known about how clinicians screen for, document and monitor their motor side effects (i.e. parkinsonism, akathisia, dystonia and tardive dyskinesia (TD), collectively “extrapyramidal symptoms” or EPS).

**Objectives:**

This review aims to systematically assess the literature for insights into current trends in EPS monitoring practices within various mental health settings globally.

**Methods:**

In line with our preregistered protocol (PROSPERO: CRD42023482372), we systematically searched the OVID Medline, PubMed, Embase, CINAHL and PsycINFO databases for studies published from 1998 to present day. Figure 1 shows a detailed flowchart of the selection process. Included studies were assessed for quality using a modified version of the Quality Improvement Minimum Criteria Set (QI-MQCS) and findings summarized using narrative synthesis. All stages of the review process are reported in accordance with the Preferred Reporting Items for Systematic Reviews and Meta-Analysis (PRISMA) guidelines.

**Results:**

A total of 22 studies met our inclusion criteria. Studies occurred in varied settings and employed a range of study designs. The APA and NICE guidelines were most commonly used to guide practice. Baseline monitoring rates in adult settings ranged from 0 to 54%, and 3.7 to 100% in child & adolescent settings. In studies reassessing EPS monitoring rates following practice improvement initiatives, virtually all demonstrated benefits. Screening processes and instruments varied, ranging from standardized rating scales (such as the AIMS for TD screening) to locally developed tools. In some studies, no structured tool was identified. Monitoring rates were higher when structured processes and tools were used.

**Image 1:**

**Figure:**
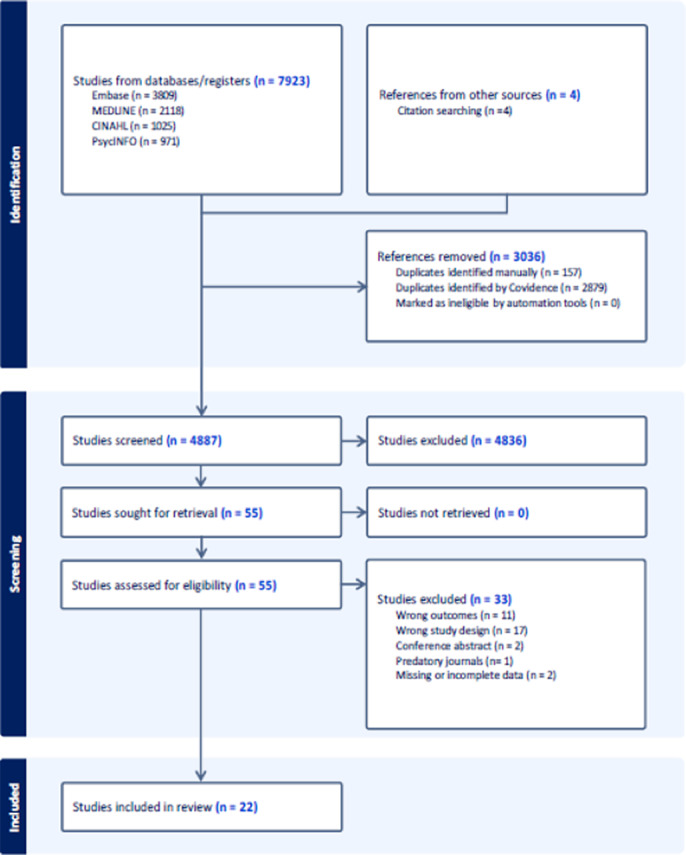

**Conclusions:**

This review demonstrates significant heterogeneity in clinical practice for the screening, documentation, and monitoring of EPS in patients prescribed antipsychotic medication in mental health settings globally. Adherence to existing guidelines was found to be poor in most settings, with practice improvements observed in virtually all settings where quality improvement initiatives were implemented. The best improvements were seen to occur after services introduced structured EPS screening tools with regular education on their use.

**Disclosure of Interest:**

R. Aubry: None Declared, T. Hastings: None Declared, M. Morgan: None Declared, J. Hastings: None Declared, M. Bolton: None Declared, M. Grummell: None Declared, R. Shorr: None Declared, S. Killeen: None Declared, C. Coyne: None Declared, M. Solmi Consultant of: MS has received honoraria/has been a consultant for AbbVie, Angelini, Lundbeck, Otsuka.

